# Supplementary material for: MetaQTL: a package of new computational methods for the meta-analysis of QTL mapping experiments
Source: BMC Bioinformatics. 2007 Feb 8;8:49. doi: 10.1186/1471-2105-8-49 (PMC1808479; doi:10.1186/1471-2105-8-49)
Supplement: Additional File 7 — MetaQTL Package : jar file and tutorial. This Zip archive contains both the MetaQTL JAR file and the files of the tutorial. [file 1471-2105-8-49-S7.zip › org.inra.metaqtl/doc/org/metaqtl/bio/IBioOntologyTerm.html]

IBioOntologyTerm


|  |  |  |
| --- | --- | --- |
| **Overview** \| **Package** \| **Class** \| **Use** \| **Tree** \| **Deprecated** \| **Index** \| **Help** | |  |
| **PREV CLASS**   **NEXT CLASS** | **FRAMES**    **NO FRAMES**     **All Classes** |  |
| SUMMARY: NESTED \| FIELD \| CONSTR \| METHOD | DETAIL: FIELD \| CONSTR \| METHOD |  |


---


## org.metaqtl.bio Interface IBioOntologyTerm

**All Superinterfaces:**: IBioAdaptable, IBioEntity

**All Known Implementing Classes:**: OntologyTerm

---

``` public interface IBioOntologyTerm extends IBioEntity ```

This class defines a term of an ontology.

---

| **Method Summary** | |
| --- | --- |
| `IBioOntologyTerm[]` | `getChildrenTerm()`             Returns the children if the term has chilren, null otherwise. |
| `long` | `getID()`             Returns the id if the terms. |
| `IBioOntologyTerm` | `getParentTerm()`             Returns the parent of the term. |
| `boolean` | `hasChildren()`             Returns true if the term has children, false otherwise. |

**Methods inherited from interface org.metaqtl.bio.IBioEntity**

`getName, getParent, getProperties, getType, setName, setProperties`

**Methods inherited from interface org.metaqtl.bio.IBioAdaptable**

`getBioAdapter`

**Method Detail**

### getID

```
long getID()
```

:   Returns the id if the terms.

    :   **Returns:**

---


### getChildrenTerm

```
IBioOntologyTerm[] getChildrenTerm()
```

:   Returns the children if the term has chilren,
    null otherwise.

    :   **Returns:**

---


### getParentTerm

```
IBioOntologyTerm getParentTerm()
```

:   Returns the parent of the term.

    :   **Returns:**

---


### hasChildren

```
boolean hasChildren()
```

:   Returns true if the term has children, false
    otherwise.

    :   **Returns:**


---


|  |  |  |
| --- | --- | --- |
| **Overview** \| **Package** \| **Class** \| **Use** \| **Tree** \| **Deprecated** \| **Index** \| **Help** | |  |
| **PREV CLASS**   **NEXT CLASS** | **FRAMES**    **NO FRAMES**     **All Classes** |  |
| SUMMARY: NESTED \| FIELD \| CONSTR \| METHOD | DETAIL: FIELD \| CONSTR \| METHOD |  |


---
